# Computational Neuropsychiatry – Schizophrenia as a Cognitive Brain Network Disorder

**DOI:** 10.3389/fpsyt.2014.00030

**Published:** 2014-03-25

**Authors:** Maria R. Dauvermann, Heather C. Whalley, André Schmidt, Graham L. Lee, Liana Romaniuk, Neil Roberts, Eve C. Johnstone, Stephen M. Lawrie, Thomas W. J. Moorhead

**Affiliations:** ^1^Division of Psychiatry, Royal Edinburgh Hospital, University of Edinburgh, Edinburgh, UK; ^2^Department of Psychiatry, University of Basel, Basel, Switzerland; ^3^Medical Image Analysis Center, University Hospital Basel, Basel, Switzerland; ^4^McGovern Institute for Brain Research, Massachusetts Institute of Technology, Cambridge, MA, USA; ^5^Clinical Research Imaging Centre, QMRI, University of Edinburgh, Edinburgh, UK

**Keywords:** computational neuropsychiatry, schizophrenia, fMRI, dynamic causal modeling, cognition, neurotransmitter, dopamine, glutamate

## Abstract

Computational modeling of functional brain networks in fMRI data has advanced the understanding of higher cognitive function. It is hypothesized that functional networks mediating higher cognitive processes are disrupted in people with schizophrenia. In this article, we review studies that applied measures of functional and effective connectivity to fMRI data during cognitive tasks, in particular working memory fMRI studies. We provide a conceptual summary of the main findings in fMRI data and their relationship with neurotransmitter systems, which are known to be altered in individuals with schizophrenia. We consider possible developments in computational neuropsychiatry, which are likely to further our understanding of how key functional networks are altered in schizophrenia.

## Introduction

Schizophrenia is a severe psychiatric disorder, which is initially manifested through positive symptoms including delusions, hallucinations, and disorganized thoughts. As the illness progresses negative symptoms such as avolition, alogia, and apathy may occur. Prior to diagnosis of illness, cognitive deficits can occur and illness progression can also be associated with cognitive deficits (1, 2). It is widely established that such cognitive deficits are considered a core symptom of schizophrenia (3) and are associated with reductions in working memory performance. Working memory deficits are one of the main neurocognitive impairments found in subjects with first episode schizophrenia (FES) (4, 5) and in people with established schizophrenia (EST) (6). Similar deficits also occur in individuals at high risk of schizophrenia [HR; Ref. (2)]. Furthermore, recent evidence has been presented, which indicates a relationship between severity of working memory deficits and the severity of negative symptoms (7). The severity of working memory deficits that is evident at the first episode of schizophrenia can predict the quality of life at the established stage of the illness (8, 9).

Two major neurotransmitter circuits have been implicated in clinical and cognitive symptoms in subjects with schizophrenia: these are the dopamine and glutamate neurotransmitter circuits. Evidence has been presented for separate alterations/disruptions of dopamine and glutamate as well as an interactive role between both neurotransmitters^1^. The two main neurobiological hypotheses in schizophrenia are based on the theories of altered dopaminergic transmission (“dopamine hypothesis of schizophrenia”) and altered glutamatergic transmission (“glutamate hypothesis of schizophrenia”). It is thought that both dopamine and glutamate modulate the dorsolateral prefrontal cortex (DLPFC) and in schizophrenia alter the performance in cognitive processes such as in working memory (10–13). Such work supports the notion of schizophrenia as a brain disorder. FMRI and positron emission tomography (PET) findings of altered functional activation and functional connectivity (FC) during working memory have been reported in people with schizophrenia when they are compared to healthy controls (14, 15). Furthermore, PET studies have presented evidence for indirect markers of altered dopamine transmission, which was correlated with working memory performance (2, 16). Alterations of indirect measures of glutamate concentrations have been reported by proton magnetic resonance spectroscopy (MRS) studies (17).

One subfield within the emerging field of computational neuropsychiatry is based on modeling fMRI networks and the evidence of (i) altered dopaminergic and/or glutamatergic transmission in (ii) cognitive function (i.e., working memory) in people with schizophrenia. Therefore, the objectives are the investigation of impaired cognitive function mediated by large-scale networks in combination with underlying neurobiological circuits such as dopamine and glutamate. Researchers in computational neuropsychiatry examine and model altered cognitive brain function in terms of functionally integrated regions [i.e., effective connectivity (EC)] (18), which may be mediated by genetic factors and neurotransmitter circuits (19–21). Mechanistic responses can be inferred from the computational modeling of cognitive brain function where the localized brain function is monitored through the BOLD response (22). This modeling approach allows computational neuropsychiatry to further our understanding of the neurobiological processes, which underlie altered cognitive brain function in individuals with schizophrenia. Thus, advancing our knowledge of schizophrenia as a cognitive brain network disorder.

In this review, we summarize fMRI findings in verbal/numeric working memory^2^ in context of (i) the understanding of schizophrenia as a cognitive brain disorder (from clinical and cognitive neurosciences) and (ii) the understanding of schizophrenia as a cognitive brain network disorder (from computational neuropsychiatry). We discuss these sets of findings in context of the dopamine and the glutamate hypotheses of schizophrenia. We consider two key research questions for the discussion of each set of findings:

(i) To what extent do these sets of findings support the dopamine hypothesis and/or the glutamate hypothesis in subjects with schizophrenia?
(ii) Do the findings from computational neuropsychiatry lead to a better understanding of schizophrenia than that obtained from clinical and cognitive neurosciences?

The review is structured as followed: first, the dopamine and glutamate hypotheses of schizophrenia are summarized (Section Schizophrenia as a Brain Disorder). Second, exemplary findings of verbal/numeric working memory deficits from fMRI studies in subjects with schizophrenia are summarized. These findings are discussed in context of the dopamine hypothesis and the glutamate hypothesis of schizophrenia (Section Schizophrenia as a Cognitive Brain Disorder). Third, we present a brief introduction to computational neuropsychiatry. We provide examples from computational neuropsychiatry and the application to the investigation of cognitive brain large-scale networks in people with schizophrenia^3^. Finally, we consider current methodological limitations of the methods (Section From Computational Neuropsychiatry Towards Schizophrenia as a Cognitive Brain Network Disorder). We outline potential future influences of computational advances in schizophrenia that may shape our understanding of schizophrenia with the aim of developing more effective treatments for the disorder (Section Understanding of Schizophrenia).

## Schizophrenia as a Brain Disorder

Neurobiological research into alterations of dopaminergic and/or glutamatergic neurotransmission has paved the way for the understanding of schizophrenia as a disorder of the brain. The dopamine hypothesis posits that dopamine function is altered in schizophrenia and that this dysfunction may be the pathophysiological pathway leading to clinical and cognitive symptoms (23, 24). The glutamate hypothesis proposes that the altered dopaminergic dysfunction may be secondary to aberrant glutamatergic dysregulation, which may contribute to clinical and cognitive symptoms in schizophrenia (25–27).

### Dopamine hypothesis of schizophrenia

The origin of the dopamine hypothesis of schizophrenia is based on the discovery of antipsychotic drugs by Delay et al. (28) in 1952. Carlsson and Lindqvit reported the first findings of an effect of antipsychotic drugs on the metabolism of dopamine (29). The dopamine hypothesis posits that alterations of dopaminergic receptors may underlie the clinical symptoms of schizophrenia (30). Over last three decades, the dopamine hypothesis of schizophrenia has undergone reformulations in light of newly available preclinical and clinical findings. Here, we consider the three main hypotheses: (i) the “dopamine receptor hypothesis,” (ii) the “modified dopamine hypothesis of schizophrenia,” and (iii) the “dopamine hypothesis: version III.”

The dopamine receptor hypothesis goes back to studies reporting antipsychotics affecting the affinity of dopamine receptors (31–33). Further evidence for the hypothesis was presented with increased synaptic monoamine levels during the induction of psychotic symptoms (34). The focus of this hypothesis rests on the excess of dopamine receptors. Thus, the clinical treatment is aimed at blocking the dopamine D_2_ subtype of the dopamine receptors (35).

The modified dopamine hypothesis of schizophrenia has been formulated to integrate new findings (36). Preclinical and clinical studies (i.e., post-mortem, metabolite, and dopamine receptor neuroimaging studies) have advanced the understanding of relationships between affinity and occupancy of D_2_ and D_1_ subtypes of the dopamine receptors and regional specificity (37). Furthermore, it was assumed that findings of altered regional dopaminergic receptor function from preclinical and indirect clinical studies could be linked to clinical symptomatology in schizophrenia (36). The hypothesis suggests that “hypofrontality,” as measured with reduced regional cerebral blood flow (rCBF) in the PFC may indicate low dopamine levels in the PFC (36). Findings from preclinical lesion studies proposed that prefrontal “hypodopaminergia” lead to striatal “hyperdopaminergia” (38, 39). In addition, it is hypothesized that prefrontal “hypodopaminergia” could cause negative symptoms, whereas striatal “hyperdopaminergia” could lead to positive symptoms (36).

The dopamine hypothesis: version III synthesizes published findings on dopamine and its potential role in schizophrenia from the main fields into one unifying hypothesis. The hypothesis aims to provide a framework for findings from developments in clinical research into genetic (risk) factors, environmental risk factors, neurochemical and neuroimaging studies, and preclinical studies, which may be related to increased presynaptic striatal dopaminergic function in schizophrenia (23). The hypothesis is comprised of four components: (i) The interaction of “hits” such as fronto-temporal dysfunction, genes, stress, and drugs may lead to striatal dopamine dysregulation (i.e., increased presynaptic dopamine synthesis capacity) and therefore to psychosis. (ii) It is hypothesized that the primary dopaminergic dysfunction is located at the presynaptic dopaminergic level instead of the D_2_ receptor level. (iii) The hypothesis assumes that the dopamine dysregulation combined with cultural and societal factors could lead to future clinical diagnosis of “psychosis” rather than schizophrenia. (iv) It is proposed that the dopamine dysfunction could change the perception and judgment of stimuli (possibly through aberrant salience), which could result in cognitive deficits (40, 41).

Recent meta-analyses, which examined markers of striatal dopamine alterations in schizophrenia, reported evidence of different types of elevated dopamine dysfunction. Supporting evidence for the dopamine hypothesis has been shown by increased striatal presynaptic dopaminergic function in medication-free or medication-naïve patients with schizophrenia contrasted to healthy controls (42) and increased striatal dopamine synthesis capacity (43). Contradictory findings have however been reported by Fusar-Poli and Meyer-Lindenberg (44), who found no difference in striatal dopamine active transporter density between patients with schizophrenia and healthy controls.

In summary, while both the dopamine receptor hypothesis and the modified dopamine hypothesis of schizophrenia have their origins in the neurobiological investigation of the mode of action of antipsychotics, the dopamine hypothesis: version III aims at integrating advances in research of schizophrenia into one unifying dopamine hypothesis. The scope of understanding of dopaminergic dysregulation has become more defined, ranging from the whole brain perspective, through the perspective of regional specificity between (DL)PFC and striatum, to the current perspective of elevated presynaptic striatal dopaminergic function. The development of the dopamine hypothesis over the three versions has helped shape the understanding of schizophrenia as a brain disorder.

### Glutamate hypothesis of schizophrenia

The origin of the glutamate hypothesis of schizophrenia was based on the discovery of psychotomimetic effects of ketamine and phencyclidine, which elicited psychotic symptoms in healthy people. Symptoms such as delusions and hallucinations experienced by healthy individuals were compared to positive symptoms seen in FES (45, 46). The glutamate hypothesis postulates a mechanistic process of altered interacting glutamatergic and/or dopaminergic neurotransmitter circuitries implicated in the pathophysiology of clinical and cognitive symptoms in schizophrenia (47–50). In this review, we consider three models of the glutamate hypothesis with relevance to the investigation of altered working memory function in people with schizophrenia: (i) the “*N*-Methyl-d-aspartate acid (NMDA) receptor hypofunction model” of schizophrenia, (ii) the “acute ketamine model,” and (iii) the “dysconnection hypothesis” of schizophrenia.

The NMDA receptor hypofunction model of schizophrenia posits that the subtype of the glutamate receptor is implicated in multiple pathological brain mechanisms of schizophrenia ranging across cellular, chemical, and neuronal levels (51–54). It has been proposed that NMDA receptor hypofunction could underlie the pathophysiology of negative and cognitive symptoms in schizophrenia (29, 51, 55, 56). Clinical trials with agents modulating NMDA receptor in addition to treatment with first-generation antipsychotics (FGA; such as chlorpromazine, haloperidol, perphenazine) and second-generation antipsychotics (SGA; such as clozapine and olanzapine) presented supporting evidence for amelioration of negative and cognitive symptoms (51, 57, 58). Evidence for the involvement of NMDA receptor hypofunction through interactions among different neurotransmitters such as γ-aminobutyric acid (GABAergic) interneurons (51) and dopamine (59, 60) has also been reported.

Evidence for the glutamate hypothesis in humans is based on clinical studies with ketamine in healthy subjects. Results suggest that glutamatergic alterations could explain the pathophysiological mechanisms resulting in positive symptoms predominantly experienced by FES and those with first episode psychosis (FEP) (45, 61). While findings from ketamine injection studies have aided the understanding of glutamatergic signaling in the development of delusions and hallucinations, evidence for altered glutamatergic transmission in negative and cognitive symptoms is scarce. FMRI findings from ketamine studies in healthy subjects propose that altered glutamatergic signaling could be implicated in working memory (12, 45, 62). These findings are in keeping with evidence from glutamatergic animal models, which report aberrant working memory function after the inhibition of glutamatergic receptors (63–66).

The dysconnection hypothesis of schizophrenia posits that altered NMDA receptor-mediated synaptic plasticity may be the underlying pathophysiological mechanism in individuals with schizophrenia (20, 21, 67). The authors propose that altered synaptic plasticity may explain both clinical symptoms and cognitive deficits in people with schizophrenia neurobiologically by altered NMDA receptor neuromodulation. Therefore, the dysconnection hypothesis synthesizes neurobiological findings (i.e., dopamine as one of the main neuromodulators leading to aberrant NMDA receptor function) with clinical and cognitive neuroscientific findings (i.e., cognitive impairment) in individuals with schizophrenia. One of the main objectives of the dysconnection hypothesis is to offer a new approach and therefore new interpretation of neurophysiological and neuroimaging data. This may be used to assist in the understanding of altered cognitive function in people with schizophrenia. For functional neuroimaging data, the biophysical modeling approach of dynamic causal modeling [DCM; Ref. (18)] has been proposed to infer biophysical processes (namely, NMDA receptor-dependent synaptic plasticity) underlying the blood-oxygen-level-dependent (BOLD) responses. In addition, the authors provide arguments that the development of positive symptoms such as delusions can be explained by a “failure of self-monitoring mechanism” or “corollary discharge” (20). Abnormal EC findings from EEG and fMRI studies across a range of cognitive tasks in subjects with schizophrenia in contrast to healthy controls have been reported (68–70). These lead to a new insight into altered connectivity above those provided by FC studies, which are formulated under different theoretical frameworks, specifically DCM findings enable the inference of biophysical processes underlying neural responses (18, 19, 71).

In summary, the three hypotheses, the NMDA receptor hypofunction model, the acute ketamine model, and the dysconnection hypothesis, have motivated researchers to investigate biophysical circuit processes implicated in glutamatergic and dopaminergic interaction in negative symptoms and cognitive function in schizophrenia. These circuit mechanisms are thought to underlie altered working memory function in schizophrenia. Research on the NMDA receptor hypofunction model has its roots in the pharmacological examination of antipsychotics, the development of new agents, and its effects on clinical and cognitive symptoms in preclinical and clinical research in schizophrenia. The focus of researchers examining the acute ketamine model and the dysconnection hypothesis lies on elucidating proposed neurobiological processes of blockade of NMDA receptor underlying altered cognitive brain function in schizophrenia. The study designs of both versions differ in the investigation of (i) the pharmacological effect of ketamine on altered cognitive brain function and clinical symptomatology in healthy controls (the acute ketamine model) and (ii) altered synaptic plasticity during altered cognitive brain function in subjects with schizophrenia. Despite the different approaches, researchers of both versions of the glutamate hypothesis share the common aim of increasing our insight into schizophrenia by the translation of neurobiological knowledge from basic research to clinical research in schizophrenia. Furthermore, researchers share the common methodological approach of large-scale network analysis of fMRI data. Taken together, development over the three versions of the glutamate hypothesis of schizophrenia have presented promising evidence for shaping the understanding of schizophrenia as a cognitive brain network disorder.

## Schizophrenia as a Cognitive Brain Disorder

Clinical and cognitive neuroscience studies have applied *in vivo* neuroimaging techniques of fMRI, PET, and single-photon emission computed tomography (SPECT) to assess neurobiological processes that underlie working memory function in people with schizophrenia. Techniques such as PET and SPECT use injections of positron-emitting radionuclide as tracer (for PET) or gamma-emitting radionuclide as tracer (for SPECT) in the living brain. Although these nuclear medical imaging techniques are non-invasive they require the administration of tracers. FMRI provides non-invasive *in vivo* imaging, which measures brain function by means of the BOLD response (72).

In the last two decades, the fields of clinical and cognitive neurosciences merged to provide a multidisciplinary approach to research into schizophrenia. This approach has created the notion of schizophrenia as a cognitive brain disorder (15, 73, 74).

### Examples of fMRI and PET studies investigating altered working memory function in subjects with schizophrenia

Working memory tasks were initially investigated with fMRI in healthy subjects (75–78). These initial findings led to the use of fMRI as a tool for examining neurobiological markers that could be related to working memory performance. The examination of working memory function was extended to individuals with schizophrenia.

Reported findings of brain function during working memory (among several domains and components of working memory tasks) in healthy controls have led to the understanding that dopamine modulates working memory in healthy controls (79–81). This evidence of dopaminergic involvement in working memory was extended by the findings of altered dopaminergic modulation in schizophrenia (74, 82). Subsequently, converging findings were reported that regions such as DLPFC, anterior cingulate cortex (ACC), and parietal cortex (PC) are activated in working memory in both healthy controls and in subjects with schizophrenia (83–86). However, in those with schizophrenia, these regions exhibit increased or reduced functional activations and FC between prefrontal and parietal regions as well as between prefrontal and temporal regions in contrast to healthy controls. Alterations in FC occur at all stages of the illness (87, 88): (i) in HR subjects (89); (ii) in FES and FEP (90), and (iii) in subjects with EST (91).

Systematic reviews and meta-analyses of working memory fMRI studies in people with schizophrenia do not report consistent findings (92–95). Some studies report increased activation of the DLPFC, commonly referred to as “hyperfrontality,” however, others report decreased activation or “hypofrontality.” This picture of differing functional activation in terms of the direction, extent, and/or pattern of BOLD responses was attributed to the variation of domains and components of working memory tasks (92–95). Also it was considered that methodological factors in the applied analyses would contribute to these variations in functional activation (93, 95, 96). In addition, differences in medication could contribute to variation in the reported functional activation between studies.

Here, we review exemplary fMRI studies using the numeric or verbal “N-back” task in subjects with EST and healthy controls, which reported functional activation and FC findings (Table 1). The reviewed studies present group differences between subjects with schizophrenia and healthy controls. In functional activation studies, evidence was reported for increased activation in DLPFC, PFC, ventral PFC, medial frontal gyrus, and AC during high working memory load in subjects with EST (89, 97–101). However, reduced activation in prefrontal regions, such as ventral PFC, DLPFC, AC, and parietal regions was found during high working memory load in subjects with EST (97, 98, 102). One study in FES found a reduction of activation in inferior frontal gyrus (IFG), superior frontal gyrus, and AC during high working memory load (103). We note three factors, which contributed to difficulties in comparing the findings across the reviewed studies: (i) missing information of phase of schizophrenia (100), (ii) heterogeneous groups of subjects with EST (97, 101, 103), and (iii) limited information on antipsychotic treatment (89, 97–101, 103). Fundamentally, none of the functional activation findings was interpreted in context of the dopamine or glutamate hypothesis. The lack of a clear understanding in terms of neural activation and pathophysiological mechanism suggests there is a need for studies examining wider prefrontal circuitry underlying working memory deficits in schizophrenia (93, 95).

**Table 1:** **Understanding of schizophrenia as a cognitive brain disorder – I summary of main findings in verbal/numeric working memory**.

Study	Subjects – phase of schizophrenia: HC – HR, FES, EST	Medication	Experimental paradigm	Functional connectivity method; seed regions/ROIs/VOIs; seed regions/ROIs/VOIs; definition; sphere size	Main finding(s)
**fMRI STUDIES – FA**					
(97)	18 HC; 13 EST^a^	Not reported	Numeric “2-back”	N/A	↑ With increasing WM load in right DLPFC, left PFC, left AC; ↓ with increasing WM load includ. right AC, right PC, left vPFC
(102)	16 HC; 17 EST	17 EST, stable injectable FGA for 2 months	Verbal “2-back”	N/A	Main effect of group: ↑ subgenual AC gyrus; group × WM load interaction for high WM load: ↓ in right DLPFC
(98)	14 HC; 14 Patients^b^ subdivided into HP: 8 HC, 7 patients; LP: 6 HC, 7 patients	14 Patients, 476.3 (291.7)^c^; 7 Patients, 556.0 (157.0)^c^; 6 Patients, 237.7 (96.4)^c^	Numeric “2-back”	N/A	↑ and ↓ for high WM load in different subdivisions of the right and left DLPFC in 14 patients; bilateral prefrontal areas of ↑ and ↓ for high WM load in HP patients; bilateral prefrontal areas of ↓ for high WM load in LP patients
(99)	22 HC; 14 EST	Not reported	Verbal “2-back”	N/A	↑ For high WM load in right medial FG; ↑ for hits during for high WM load in right medial FG
**fMRI STUDIES – FC**					
(100)	26 HC; 15 Patients^b^, subdivided into HP: 14 HC, 8 patients; LP: 12 HC, 7 patients	15 Patients, 501 (337.0)^c^	Numeric “2-back”	Seed-based cross-correlation; seed regions: right dPFC and left vPFC; functional ROIs 10 mm sphere size	↑ FA with increasing WM load in bilateral vPFC in 15 patients; ↑ FC between left vPFC and left SPL in 15 patients; ↓ FC between right dPFC and bilateral IPL in 15 patients
(89)	153 HC; 78 EST^b^	75 EST, FGA, and SGA; 3 EST, data missing	Numeric “2-back”	Seed-based cross-correlation^d^; Seed regions: right DLPFC; functional ROIs 6 mm sphere size	↑ FA for high WM load in right DLPFC; ↓ FC between right DLPFC and bilateral HF; ↓ FC between right DLPFC and right IPL
(103)	28 HC; 30 FES^a^	Not reported	Numeric “2-back”	Seed-based cross-correlation; seed regions: left gyrus rectus, left IFG, left SFG, left AC, right PHG, right amygdala; functional ROIs sphere size not reported	↑ FA for high WM load in left gyrus rectus, left IFG, left SFG, left AC, right PHG, right amygdala; ↓ FC between medial FG and right precuneus; between medial FG and left OFG; between medial frontal gyrus and right precentral gyrus
(101)	28 HC; 28 EST^a^^,^^b^	24 EST, 294.45 (316.36)^c^	Numeric “2-back”	ROI-to-ROI FC; ROIs: bilateral DLPFC, vlPFC, putamen, caudate nuclei, IPL; functional ROIs sphere size not reported	↑ FA for high WM load in bilateral putamen, left DLPFC, OFC, cuneus, and PC; ↓ FC between left putamen and right vlPFC; ↓ FC between left putamen and left vlPFC; between right IPL and right vlPFC

*↑Increased in subjects with schizophrenia in contrast to HC*.

*↓Decreased in subjects with schizophrenia in contrast to HC*.

*AC, anterior cingulate; DLPFC, dorsolateral prefrontal cortex; dPFC, dorsal prefrontal cortex; EST, subjects with established schizophrenia; FA, functional activation; FC, Functional connectivity; FES, subjects with first episode schizophrenia; FG, frontal gyrus; FGA, First-generation antipsychotics; HC, healthy controls; HF, hippocampal formation; HP, high-performers; HR, subjects at high risk of schizophrenia; IFG, inferior frontal gyrus; IPL, inferior parietal lobe; LP, low-performers; OFG, orbitofrontal gyrus; PC, posterior cingulate; PHG, parahippocampal gyrus; ROI, region of interest; SGA, second-generation antipsychotics; SFG, superior frontal gyrus; SPL, superior parietal lobule; vPFC, ventral prefrontal cortex; vlPFC, ventrolateral PFC; WM, working memory*.

*^a^Patients with different schizophrenia subtypes, such as paranoid subtype, schizoaffective subtype, undifferentiated subtype*.

*^b^Phase of illness, illness onset, and illness duration not reported. Phase of illness based on symptoms scores*.

*^c^Chlorpromazine equivalents in milligrams per day*.

*^d^Seed-based connectivity only reported here*.

Functional connectivity studies applied voxel-based seed approaches to the BOLD response (89, 100, 103), with the exception of one study, which applied an ROI-to-ROI approach (101). Despite equivalent methodological approaches, the FC findings are not entirely comparable due to the use of different seed locations. Findings of reduced connectivity involving subregions of the PFC were found in FES and EST. Reduced FC findings in subjects with schizophrenia and EST were reported in the majority of studies: (i) Reduced prefrontal–parietal^4^ FC in subjects with schizophrenia (100); (ii) Reduced prefrontal–hippocampal, prefrontal–striatal, and within-PFC FC in EST (89); and (iii) Reduced parieto-prefrontal FC and between putamen and ventrolateral PFC in EST (101). Further evidence for reduced FC between medial frontal gyrus and putamen was found in FES (103). In contrast to most studies that report reduced FC in the early and late phases of the illness, increased FC between the ventral PFC and posterior PC was shown in subjects with schizophrenia (100). The findings of both reduced and increased FC between subregions of the PFC and the posterior PC may be related to variations in behavioral response to task load for subjects with schizophrenia (100). Similar difficulties in comparing the FC findings among the studies are present as in the comparison of the functional activation studies due to unclear and missing information regarding the illness phase, diagnosis, and medication treatment. Similarly, no reference is made to the dopamine or glutamate hypothesis in interpreting the FC findings.

In summary, findings presented by FC studies during the “N-back” task have paved the way for the understanding of large-scale functional networks in working memory. Furthermore, the insight of brain alterations in subjects with schizophrenia has advanced with FC from individually activated regions to connectivity between brain regions. The perspective of circuit-based neurobiology and cognitive brain function opens the doors for translational research from preclinical and clinical research in schizophrenia. However, FC is limited as the connection assessments are based upon regional correlations and this approach does not allow inferences of directions or causality between connected regions (18).

Positron emission tomography and SPECT imaging in schizophrenia research are used to investigate indirect markers of dopamine measures such as D2/3 receptors, presynaptic dopaminergic function, dopamine synthesis capacity, dopamine release, and dopamine transporters. Three [H215O] PET studies consistently reported reduced rCBF in DLPFC and PC in verbal/numeric “2-back” in subjects with EST in contrast to healthy controls (104–106). Reduced prefrontal–hippocampal FC findings in subjects with schizophrenia in contrast to healthy controls (105, 106) confirmed the hypothesis of reduced functional connections in working memory. Correlational PET studies provided support for dopaminergic alterations and measures of the “2-back” task in subjects with schizophrenia (2, 16).

**Figure 1:**
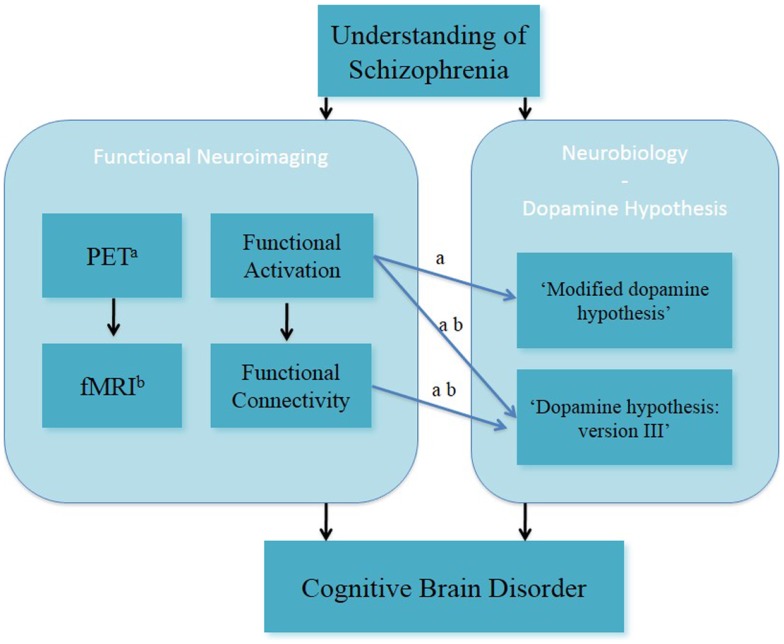
**Understanding of schizophrenia as a cognitive brain disorder – verbal/numeric “N-back” task**. ^a^fMRI; ^b^PET, positron emission tomography.

In summary, fMRI and PET studies in the field of clinical and cognitive neurosciences have been used to investigate brain function during working memory in people with schizophrenia (Figure 1). Both fMRI and PET findings have advanced the understanding of altered working memory performance and brain function in subjects with schizophrenia. This has led to better insight into the interaction between altered working memory function and experimental/clinical factors (such as cognitive domains of working memory function, performance level, phases of illness, clinical symptomatology, and effects of antipsychotic medication) in individuals with schizophrenia.

### Examples of fMRI studies investigating altered spatial working memory function – glutamate hypothesis of schizophrenia

The role of the DLPFC in working memory deficits has been associated with glutamatergic alterations and more specifically in dopamine–glutamate interactions (10, 50, 51). Furthermore, it has been reported that ketamine, a NMDA receptor antagonist, can induce psychosis-like symptoms in healthy subjects (45). Here, we briefly summarize the main functional activation and FC findings of fMRI studies on the spatial “N-back” task in the context of the glutamate hypothesis of schizophrenia (Table 2).

**Table 2:** **Schizophrenia as a cognitive brain disorder II – summary of main findings in spatial working memory**.

Study	Subjects HC	Medication/ketamine injection	Experimental paradigm	Functional connectivity method	Computational modeling	Main finding(s)
				Seed regions/ROIs	
				Seed regions/ROIs definition	
				Sphere size	
**fMRI + KETAMINE STUDIES**						
(12)	19 HC	One saline injection; initial ketamine bolus 0.23 mg/kg for I min; subsequent ketamine bolus 0.58 mg/kg for 1 h	Spatial “2-back” and “4-back”	Seed-based cross-correlation	Modeling of the acute ketamine effect of local and long-range E–I connections	Ketamine attenuated task-activated regions (i.e., DLPFC and precuneus)
				Seeds: FP–DMN pair; CO–DMN pair; anatomical ROIs 15 mm sphere size	Spiking E and I cell local-circuit models: task-activated module and task-deactivated module	Ketamine attenuated task-deactivated regions overlapping the DMN; ↓ E–I conductance led to attenuation in task-activated regions; modulation of task-activated FC between FP–DMN networks during delay of WM
(62)	22 HC	One saline injection; one initial ketamine bolus 0.23 mg/kg for 1 min; one subsequent ketamine bolus 0.58 mg/kg for 1 h	Spatial “2-back” and “4-back”	(1) Seed-based cross-correlation; seed regions: bilateral MFG, IFG, SFG, and Heschl’s gyrus; anatomical ROIs 10 mm sphere size	N/A	Ketamine effect using (1): ↓ FC between right DLPFC and MFG, IFG, frontal OC, insula, medial FG; angular gyrus; ketamine effect using (2): ↓ FC within left DLPFC
				(2) Global-based connectivity; see details as in (1)	

*↓Reduced*.

*CO, cingulo-occipital; DLPFC, dorsolateral prefrontal cortex; DMN, default-mode network; E cells, excitatory cells; FC, functional connectivity; FG, frontal gyrus; FP, fronto-parietal; HC, healthy controls; I cells, inhibitory cells; IFG, inferior frontal gyrus; MFG, middle frontal gyrus; NMDA, *N*-Methyl-d-aspartate acid; OC, orbital cortex; ROI, region of interest; SFG, superior frontal gyrus; WM, working memory*.

Anticevic et al. (12) presented ketamine-induced reduced functional activation in task-activated regions (such as the DLPFC and the precuneus) and task-deactivated regions of the default-mode network (DMN). In addition, the combination of a spiking local-circuit model of performance during the spatial “N-back” task and the functional activation findings revealed that the modulation of ketamine alters the association between the task-activated and the task-deactivated networks. Finally, it was shown that ketamine modulates FC between the fronto-parietal and DMN networks. In a recent study, Driesen et al. (62) provided further support for ketamine-induced reduced prefrontal FC during the spatial “N-back” task. Two FC approaches with the same seed regions were employed, seed-based FC and global-based connectivity (GBC), which revealed both decreased FC within the DLPFC. The seed-based analysis resulted in reduced FC between DLPFC and middle frontal gyrus [MFG, IFG, and insula (among other regions) under ketamine in contrast to saline]. The GBC analysis showed decreased FC of the DLPFC under ketamine.

**Figure 2:**
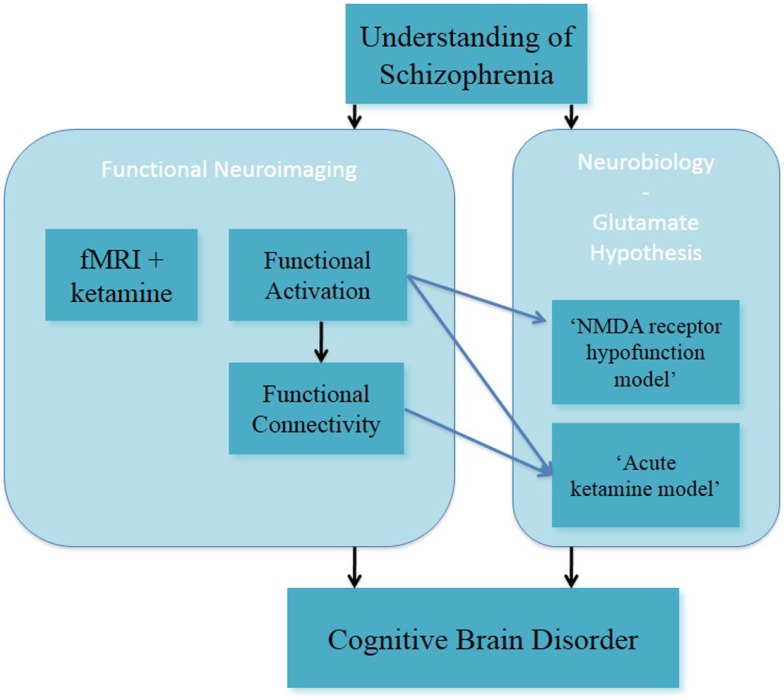
**Understanding of schizophrenia as a cognitive brain disorder – spatial “N-back” task**.

In summary, these studies on altered spatial working memory function inform on the glutamate hypothesis, through the acute ketamine model (Figure 2). In this, they have advanced the understanding of NMDA receptor-modulated brain function in healthy subjects.

## From Computational Neuropsychiatry toward Schizophrenia as a Cognitive Brain Network Disorder

Clinical and cognitive neurosciences have advanced the understanding of altered working memory function in subjects with schizophrenia. FMRI studies in working memory among other neuroimaging and electrophysiological techniques, have reported on functional activation and FC findings in subjects with schizophrenia. Both findings of functional activation and FC revealed methodological, cognitive, and clinical factors related to our understanding of altered working memory function in patients with schizophrenia. In particular, FC findings mark the beginning of the notion of “disconnection” and “dysconnection” (20, 21, 67, 107) in working with people with schizophrenia. FC is defined as the statistical association or dependency among two or more anatomically distinct time-series (107). FC findings cannot be interpreted in terms of causal effects between connected regions and thus, does not allow for a mechanistic inference of the BOLD responses.

The modeling of functional large-scale networks^5^ during working memory function in schizophrenia could provide mechanistic explanations for altered brain function in individuals with schizophrenia. The advantage of modeling functional large-scale networks in terms of EC over FC is that inferences can be drawn on mechanistic processes, which are not directly observable in the BOLD response.

### Computational neuroscience and computational neuropsychiatry

Marr proposes a theoretical framework for computational research on the brain on three levels (1976). At the first level, researchers should aim to gain knowledge of the high-level computations of the brain such as working memory (“computational level”). At the next level, the testing of the brain’s methods and algorithms for the high-level working memory function is led by hypotheses derived from the acquired knowledge and testing how appropriate an algorithm such as Bayesian inference is for modeling the working memory brain function (“algorithmic level”). Finally, when an algorithm is found, which is valid and more likely than alternative algorithms to predict known brain function/behavior, then the investigation of the biological implementation can be pursued (“physical level”).

Computational neuropsychiatry is an emerging field within computational neuroscience. Computational neuropsychiatry aims to provide an explanatory bridge between altered cognitive function and neurobiological mechanisms associated with the development of mental illness (108, 109, Huys, unreferred preprint). Computational neuropsychiatry in humans has been defined by outlining a set of components, which include biophysical modeling and computational modeling (109). Different types of computational models at different neural levels are used dependent on the study hypothesis (108, Huys, unreferred preprint).

### Computational neuropsychiatry and modeling of functional large-scale networks in subjects with schizophrenia

Connectionist and neural network models in working memory/cognitive control in subjects with schizophrenia have added to our understanding of both the brain function and the neurobiological mechanism underlying working memory (74, 76). The strength of these models is based on the translational link between human brain function (i.e., functional activation) and preclinical neurobiological evidence (namely, dopaminergic modulation) during working memory.

Following on from the work of Cohen and Braver, evidence for the understanding of schizophrenia as a cognitive network disorder has been presented by both preclinical studies (8, 10, 110–113) and human FC studies in working memory (89, 100, 101, 103). Recent studies examining biophysical mechanisms underlying altered functional large-scale networks aim to bridge the gap between the human functional network used in working memory and the preclinical neurobiological processes. Examples of such computational neuropsychiatric studies, including EC during working memory in subjects with schizophrenia, are reviewed. In this, we focus on DCM studies investigating the numeric/verbal “N-back” task in subjects with schizophrenia and healthy controls. This is considered in the context of the dopamine and glutamate hypotheses of schizophrenia. Both neurobiological hypotheses have contributed to the formulation of research objectives in computational neuropsychiatry (114) and the development of computational modeling techniques of fMRI data in subjects with schizophrenia (20).

#### Dynamic causal modeling for fMRI – examples of modeling functional large-scale networks

Dynamic causal modeling for fMRI has been introduced as a method to provide insight into the notion of “functional integration” during cognitive performance. “Functional integration” has been advanced from the historic notion of “functional specialization” (115), which is defined by context-dependent interactions among different brain regions (18, 116–118).

Dynamic causal modeling has been described as a biophysical modeling of neuronal dynamic processes (18, 19)^6^ , which can be used as a method for the computation of synaptic plasticity from fMRI task-based studies (20, 21). Together biophysical modeling and Bayesian inference analysis form the framework for DCM (71, 117, 118). Thus, DCM is a modeling approach, which combines defined network models (i.e., hypotheses) with Bayesian inversion methods (19, 117). Specifically, DCM assesses inter-regional EC through assessment of experimentally induced changes (18) and therefore allows for mechanistic inferences from neuronal function.

Bilinear DCM infers dynamics at the neuronal level by translating modeled neuronal responses into predicted BOLD measurements (18). Non-linear DCM for fMRI (71, 119) is an advanced approach for increasing the biological plausibility of DCMs by the means of modeling “gain modulation” (i.e., non-linear modulation of neuronal connections) (19, 117, 118). In non-linear DCM, the modulation of connection strengths by experimental inputs is supplemented by direct modulation of neural activity in one or more network regions (18, 119). The computations for gating in neural networks use the multiplicative computation of non-linear modulation (120, 121). Accordingly, non-linear DCM can be used for inferring that the strength of a connection is modulated by activity of other neuronal populations (119, 122).

##### Findings of altered effective connectivity during working memory in subjects with schizophrenia

The first DCM studies in healthy controls described large-scale networks in working memory and a similar task [continuous performance test; (123–125)]. A recent study in healthy controls built the linkage between EC results and underlying dopaminergic modulation of large-scale networks comprising of the DLPFC and PC during verbal memory performance (126).

To date four DCM studies have examined the verbal/numeric “N-back” task in subjects with schizophrenia using bilinear DCM (127–130) (Table 3). These provide novel insights into reduced task-dependent EC and increased task-independent EC measures through modeling large-scale networks in schizophrenia.

**Table 3:** **Schizophrenia as a cognitive brain network disorder II – summary of main findings in verbal/numeric working memory – neuroimaging and biophysical modeling**.

Study	Subjects – phase of schizophrenia; HC – HR, FES/FEP, EST	Medication	Experimental paradigm	Networks – model space; number of models; regions	DCM settings – DCM version; sphere size; inference technique(s)	Main finding(s)
(127)	13 HC; 16 HR^a^; 10 FES	HR, not medicated; 7 FES, risperidone or quetiapine, 3 FES, not medicated	Numeric “2-back”	1 Left hemispheric model; STG, SMA, MFG, INS, PPC	DCM in SPM5; Sphere sizes not reported; BMS not performed	Progressively ↑IC of the prefrontal–temporal connection in HR and FES
(128)	42 HC; 41 EST	35 EST, FGA; 5 EST, SGA; 1 EST, not medicated	Numeric “2-back”	48 Intrahemispheric models; 3 model families; DLPFC, PC, VC	DCM10 in SPM8; 4 mm spheres; random-effects BMS^e^^,^^f^; BMA	↓EC (effect of task-modulation) from DLPFC to PC
(129)	20 HC; 17 HR^a^; 21 FEP	HR, not medicated; 7 FEP, completely antipsychotic naïve; 6 FEP, antipsychotic naïve at the time of scanning; 8 FEP, SGA	Verbal “2-back”	12 Intrahemispheric models; bilateral SPL, bilateral MFG	DCM10 in SPM8; 12 mm spheres; random-effects BMS^e^; BMA	Progressively ↓EC (effect of task-modulation) between MFG and SPL in HC, HR, and FES. Ameliorated EC correlated with antipsychotic treatment.
(130)	15 HC; 14 FES^b^; 19 FES^c^	FES^b^, 254.76 (192.09)^d^; FES^c^, 325.88 (185.19)^d^	Verbal “2-back”	5 Left hemispheric models; medial PFC, PCC	DCM version not reported; SPM8; 6 mm spheres; BMS^e^; BMA	↑IC from PCC to medial PFC in both FES^e^ and FES^f^

*↑Increased in subjects with schizophrenia in contrast to HC*.

*↓Decreased in subjects with schizophrenia in contrast to HC*.

*BMA, Bayesian model averaging; BMS, Bayesian model selection; DLPFC, dorsolateral prefrontal cortex; EC, effective connectivity; EST, subjects with established schizophrenia; FES, subjects with first episode schizophrenia; FEP, subjects with first episode psychosis; FGA, first-generation antipsychotics; HC, healthy controls; HR, subjects at high risk of schizophrenia; IC, intrinsic connectivity; IFG, inferior frontal gyrus; INS, insula; MFG, middle frontal gyrus; PC, parietal cortex; PCC, posterior parietal cortex; PFC, prefrontal cortex; SGA, second-generation antipsychotics; SMA, supramarginal area; SPL, superior parietal lobe; STG, superior temporal gyrus; VC, visual cortex*.

*^a^Subjects at high clinical risk of schizophrenia*.

*^b^With high suicidal risk*.

*^c^With low suicidal risk*.

*^d^Chlorpromazine equivalents in mg/day*.

*^e^BMS at the group-level*.

*^f^BMS at the model family level*.

In the first study, increased fronto-temporal intrinsic connectivity was found to be associated with increased functional activation of the superior temporal gyrus (STG) during the numeric “N-back” task in the subjects at the prodromal and at the acutely psychotic stage of schizophrenia in contrast to the healthy controls. This suggests a potential marker for vulnerability to the disorder (127). Furthermore, progressively decreased intrinsic connectivity between the STG and the MFG in subjects at-risk mental state (ARMS) and FES subjects in contrast to the healthy controls was reported. This finding suggested that functional activation may resemble increased task-independent EC between the PFC and the STG. However, the results of the study are not comparable to other DCM studies because (i) only one model was examined and (ii) the biological plausibility of the EC measures is not clearly accessible. No reference to the dopamine or glutamate hypotheses was made.

The second study investigated the working memory-dependent modulatory effect for the prefrontal–parietal connectivity in subjects with EST and healthy subjects during the numeric “N-back” task (128). The large-scale networks included the right DLPFC, the PC, and the visual cortex with bidirectional connection between all regions. The main finding was decreased task-dependent EC from the DLPFC to the PC in the subjects with EST. Thus, this finding could resemble evidence for the glutamate hypothesis of schizophrenia, specifically the NMDA receptor hypofunction model and the dysconnection hypothesis.

The third study examined possible vulnerability markers for psychosis from the verbal “N-back” task in ARMS subjects, FES subjects, and healthy subjects (129). This study examined reduced task-dependent EC measures as well as relationships between connectivity parameters and antipsychotic medication received by subjects. In this study, EC in interhemispheric large-scale networks between the bilateral superior parietal lobes (SPL) and the bilateral MFG was assessed. This study reported novel findings of progressively decreased working memory and induced modulation of connectivity between the MFG and the SPL (from healthy subjects to ARMS). Additionally, further decreased EC of modulatory effects were observed in non-medicated subjects with FEP contrasted to healthy controls. Evidence for amelioration of reduced EC between the MFG and the SPL in subjects with FES, who received SGA medication, could reflect alterations of dopaminergic regulation of NMDA receptor-dependent synaptic plasticity of fronto-parietal connections. However, this interpretation is limited by the lack of a control group of FES who are treated with different types of antipsychotic medication. These findings across different subpopulations of schizophrenia together with the effect of antipsychotic medication may reflect support for the NMDA receptor hypofunction model and the dysconnection hypothesis.

In the fourth study, Zhang et al. (130) explored EC measures in terms of possible neurobiological markers in groups of subjects with schizophrenia with high or low suicide risk and contrasted these with healthy controls during the verbal “N-back” task. The large-scale networks were defined by unidirectional and bidirectional connections between the two regions of the medial PFC and PC as well as working memory effects on these regions. This pilot study presented novel findings in subjects with schizophrenia at suicidal risk in terms of increased intrinsic connectivity from the PC to the MFG in both groups with FES (in comparison to healthy controls). This finding was interpreted as a possible association to schizophrenia, in which increased intrinsic connectivity from the MFG to the PC in the subjects with high risk of suicide could reflect vulnerability of suicide. However, the results are not directly comparable to the other DCM studies because of the study population, which focused on the issue of suicide. The findings were also not interpreted in context of the dopamine or glutamate hypotheses.

We highlight main experimental and methodological limitations in the four DCM studies, which impede the comparability of findings (please see Table 3 for details). The main experimental limitation focuses on the discrepancies between the different patient subpopulations. Two studies analyzed working memory fMRI data of subjects with ARMS and FES in comparison to healthy controls (127, 129), whereas one study modeled scans from subjects with EST (128). Zhang et al. (130) reported findings of a unique patient population of FES with high and low suicidal risk. In terms of methodological issues, one limitation lies in different definitions of model spaces for the large-scale networks, despite equivalence in the experimental tasks. Another limitation is that the reviewed DCM studies employed deterministic DCM for the comparison of the models. Deterministic models can predict processes perfectly if all inputs are known (131). However, at this early stage of employing biophysical modeling approaches to human brain function, we do not have a full understanding of the brain responses to working memory. Future studies may employ stochastic DCM as an extension (117, 118, 132). A further limitation is that different DCM versions were applied across the four studies, which impede the comparability of the findings. The priors are differently defined in the used DCM versions, which give rise to a variation in model evidence between the studies (117). Thus, it is possible that discrepancies in EC findings could be due to the prior definition and may not be solely due to differences in performance, brain function, or clinical aspects of subjects with schizophrenia. Lastly, a general limitation of DCM for fMRI is that maximally 10 regions within a large-scale network can be modeled. This simplification results in difficulties of biophysical modeling of tasks, which are likely to encompass more than ten regions. Furthermore, not only the definition of different regions and different numbers of regions but also different modulatory inputs result in further extensions to the model space. Such model spaces are difficult to validate and analyze.

The four DCM studies presented evidence for increased task-dependent EC and increased task-independent EC findings during verbal/numeric working memory in subjects with schizophrenia. We discuss these EC findings in context of (i) the dopamine and glutamate hypothesis and (ii) FC findings during verbal/numeric working memory in subjects with schizophrenia.

The four studies modeled large-scale networks during the “N-back” task in subjects with schizophrenia. However, only two out of these four studies consider their DCM results in the light of biophysical processes (128, 129). The findings of reduced EC (namely, the effect of task-modulation) of the prefrontal–parietal connection in subjects with schizophrenia in contrast to healthy controls were interpreted biophysically and linked to the NMDA receptor hypofunction model and the dysconnection hypothesis (128, 129). Both studies reported reduced EC findings of the prefrontal–parietal connection during working memory, however, these findings need to be considered carefully due to different experimental designs (i.e., patient subpopulations, antipsychotic medication treatment of FGA and SGA) and methodological implementation (i.e., model space, DCM settings, and inference techniques).

Three of the DCM studies reported altered EC findings of the prefrontal–parietal and parieto-prefrontal connections during the “N-back” task in subjects with schizophrenia in contrast to healthy controls. Deserno et al. (128) and Schmidt et al. (129) presented reduced EC (effect of task-modulation) of the prefrontal–parietal connection in subjects with schizophrenia in contrast to healthy controls, whereas Zhang et al. (130) found increased EC (intrinsic connectivity) of the parietal–prefrontal connection. The reduced task-dependent EC findings are in keeping with reduced FC findings of these connections, although increased FC between a different prefrontal subregion and the PC was reported (100).

The study by Crossley et al. (127) reported increased EC (intrinsic connectivity) of the prefrontal–temporal connection in subjects at HR and FES (in contrast to healthy controls). Reduced FC of the prefrontal–temporal connection during the “N-back” task in subjects with schizophrenia has been previously reported in PET studies (105, 106). However, the regions within the PFC and temporal region differ between the studies.

##### Findings of altered effective connectivity during verbal fluency in subjects with schizophrenia

Here, we discuss bilinear and non-linear DCM studies, which have assessed large-scale networks during verbal fluency [namely, the Hayling sentence completion task (HSCT)] in subjects with schizophrenia and healthy controls (Table 4). One bilinear DCM study in healthy controls investigated the task-dependent modulation of response initiation and response suppression in EC between left hemispheric temporal and prefrontal regions (133). The main finding was a difference in connection strength of the modulatory effect in response initiation and response suppression.

**Table 4:** **Schizophrenia as a cognitive brain network disorder II – summary of main findings in verbal fluency – neuroimaging and biophysical modeling**.

Study	Subjects – phase of schizophrenia; HC – HR, FES/FEP, EST	Medication	Experimental paradigm	Networks – model space; number of models; regions	DCM settings – DCM version; sphere size; inference technique(s)	Main finding(s)
(134)	15 HC; 15 HR^a^	2 HR risperidone and quetiapine^c^	HCST	14 Left hemispheric models; MFG, ACC, MTG	DCM in SPM5; 12 mm spheres; random-effects BMS4	↑ IC between ACC and MTG; same winning model in both groups
(135)	19 HC; 26 HR^b^ – 20 HR+, 4 HRill	Not medicated	HCST	8 Left hemispheric models; 3 model families; IPS, IFG, ACC, MTG, MD thalamus	DCM8 in SPM8; 8 mm spheres (IPS, DLPFC, MTG); 6 mm spheres (ACC, MD thalamus); Random-effects BMS^d^^,^^e^; BMA	Progressively ↓ connection strength with non-linear modulation of the thalamo-cortical connection in HR+ and HRill in contrast to HC

*↑Increased in subjects with schizophrenia in contrast to HC*.

*↓Decreased in subjects with schizophrenia in contrast to HC*.

ACC, anterior cingulate cortex; ARMS, subjects with at-risk mental state; BMA, Bayesian model averaging; BMS, Bayesian model selection; EC, effective connectivity; FEP, subjects with first episode psychosis; FES, subjects with first episode schizophrenia; HC, healthy controls; HR−, subjects at high familial risk of schizophrenia without transient psychotic symptoms; HR+, subjects at high familial risk of schizophrenia with transient psychotic symptoms; HRill, subjects at familial risk of schizophrenia who subsequent to the scanning developed schizophrenia; HSCT, Hayling sentence completion task; IC, intrinsic connectivity; IFG, inferior frontal gyrus; IPS, intraparietal sulcus; MD mediodorsal; MFG, middle frontal gyrus; MTG, middle temporal gyrus;

*^a^Subjects at high clinical risk of schizophrenia*.

*^b^Subjects at high familial risk of schizophrenia*.

*^c^At the time of scanning*.

*^d^BMS at the group-level*.

*^e^BMS at the model family level*.

Two clinical bilinear DCM studies have investigated EC measures during the HSCT in HR subjects and healthy controls: (i) Subjects at high clinical risk of schizophrenia [ARMS; Ref. (134)] and (ii) subjects at high familial risk of schizophrenia (135). Allen et al. (134) investigated increased fronto-temporal EC (intrinsic connectivity) as a potential measure of vulnerability of developing schizophrenia. Two main findings were reported: firstly, no significant effect of task-dependent modulation on the fronto-temporal connection between ARMS subjects and healthy controls was revealed. Secondly, ARMS subjects displayed increased intrinsic connectivity between the ACC and the MTG in comparison to healthy controls. Furthermore, the Bayesian model selection (BMS) approach revealed that the same network was equally likely to explain the given HSCT fMRI data in both the ARMS subjects and the healthy controls. No reference to the glutamate hypothesis was made.

Dauvermann et al. (135) modeled EC measures in a similar version of the HSCT that was used by Allen et al. (134). This study was conducted in subjects at high familial risk of schizophrenia and healthy subjects. The results reported by Allen et al. (134) of a similar large-scale network in both HR subjects and healthy controls was replicated^7^. This finding was also confirmed by Dauvermann et al. (135), when the group of HR subjects was subdivided into high risk subjects without transient psychotic symptoms (referred to as HR−), high risk subjects with transient psychotic symptoms (referred to as HR+) and high risk subjects who subsequent to scanning developed schizophrenia [referred to as HRill; please see Ref. (136, 137)]. Comparability between these two studies is limited due to differences in the model space. The model space in Dauvermann et al. (135) includes the IPS and the mediodorsal thalamus, which are not incorporated in the model space by Allen et al. (134). In addition, endogenous connections and task-dependent modulations were accordingly changed [Ref. (135); Table 4]. There was no reference to the glutamate hypothesis of schizophrenia.

Limitations of bilinear DCM have been addressed through the development of non-linear DCM for fMRI (119). This method was applied in the genetic high risk study reported by Dauvermann et al. (135). The progress from the bilinear DCM to the non-linear DCM as reported by Dauvermann is based on the biophysical modeling of connection strength with non-linear modulation during the HSCT response. The authors show that relative to healthy controls there is reduced connection strength with non-linear modulation of the thalamo-cortical connection during the HSCT in HR+ subjects and a further reduction in this connection strength in HRill subjects (135). The authors suggest that reduced gain control may underlie the reduced strength in the thalamo-cortical connection. Furthermore, the findings of reduced connection strength with non-linear modulation of the thalamo-cortical connection could reflect altered glutamatergic neurotransmission, which may underlie a disruption of synaptic plasticity in this thalamo-cortical connection [Ref. (135); Table 4]. Thus, the findings were interpreted in context of the NMDA receptor hypofunction model and the dysconnection hypothesis.

#### Summary of studies modeling functional large-scale networks – dynamic causal modeling for fMRI

Evidence from brain function in working memory in subjects with schizophrenia at the level of functional large-scale networks (i.e., clinical and cognitive neurosciences) and neurobiological mechanisms in working memory in animal models of schizophrenia (preclinical neurobiological research) in combination with computational neuroscientific approaches has informed and enabled research in computational neuropsychiatry.

Exemplary DCM studies in subjects with schizophrenia have reported both increased and reduced EC findings during cognition in subjects with schizophrenia in contrast to healthy controls. These studies applied DCM as a biophysical modeling approach to functional large-scale networks, which enabled the interpretation of EC findings on the basis of the glutamate hypothesis of schizophrenia, namely the NMDA receptor hypofunction model and the dysconnection hypothesis (128, 129, 135). We emphasize that the findings support not only the glutamate hypothesis but also the dopamine hypothesis. Dopamine is a neuromodulator that may crucially affect glutamate-induced synaptic plasticity. Synaptic plasticity may be involved in a regulation of dopamine synthesis and release via other neurotransmitter systems. Specifically for non-linear effects, it has been shown that dopamine acts as a neuromodulator mediating postsynaptic gain (74, 138).

**Figure 3:**
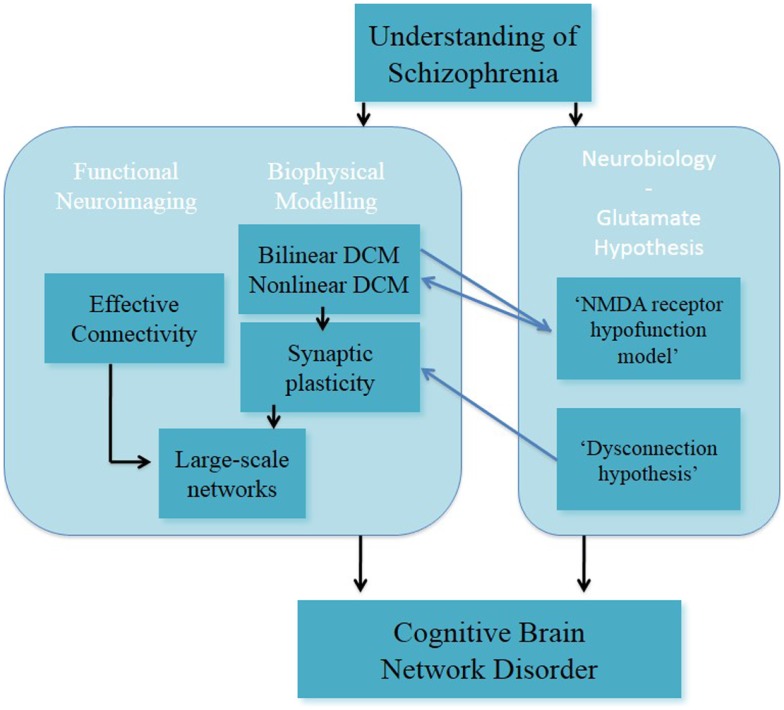
**Understanding of schizophrenia as a cognitive brain network disorder – verbal/numeric “N-back” task**.

In a recent study, it has been reported that the combination of the DCM analysis of numerical “N-back” task in EST (128) and generative embedding resulted in the dissection of three subgroups of EST based on the mechanistically inferred DCM findings (139). This exemplary study showed that DCM can be applied as a generative model of large-scale networks in individuals with schizophrenia. In summary, DCM is a promising approach for modeling synaptic plasticity; nevertheless in its current form it cannot reflect the full complexity in the processing required for the implementation of tasks such as working memory (Figure 3).

## Understanding of Schizophrenia in Development

Our understanding of schizophrenia is in continuous development and with more preclinical and clinical findings being published this understanding will advance further. A critical aspect of this understanding is the facilitation of multidisciplinary approach between preclinical and clinical research in schizophrenia.

The original understanding of schizophrenia as a brain disorder stems from observational clinical work, which led onto preclinical investigation. Over time, the knowledge of alterations of cellular, chemical, and molecular mechanisms has increased: (i) findings of dopaminergic and glutamatergic modulation of working memory (and clinical features) in animal models of schizophrenia contributed to form the understanding of schizophrenia as a cognitive brain disorder; (ii) findings of neurotransmitter circuit systems, mainly dopaminergic and glutamatergic systems, were found to modulate working memory in animal models of schizophrenia in combination with computational studies (140), which plays a role in shaping the understanding of schizophrenia as a cognitive network disorder.

Understanding of schizophrenia has not only been shaped by preclinical research but also by clinical research in subjects with schizophrenia, which has been and continues to be illuminated by preclinical neurobiological and computational work. The field of clinical and cognitive neurosciences has contributed to forming our understanding of schizophrenia as a cognitive brain disorder. Importantly, the multidisciplinary field of computational neuropsychiatry (preclinical neurobiology, clinical and cognitive neurosciences, and computational psychiatry) has allowed for progress in our understanding of schizophrenia as a cognitive brain network disorder.

### Schizophrenia as cognitive brain network disorder

The use of computational neuropsychiatric research in developing our understanding of schizophrenia as a cognitive brain network disorder is at an early stage. Here, we focused on FC and EC studies (DCM studies) during the verbal/numeric “N-back” task in subjects with schizophrenia and healthy controls. We discuss these FC and EC findings in context of two key research questions. Consideration of these questions was seen as a means to inform future schizophrenia research in the fields of clinical and cognitive neurosciences and/or computational neuropsychiatry:

#### To what extent do these sets of findings support the dopamine hypothesis and/or the glutamate hypothesis in subjects with schizophrenia?

Studies reported both increased and reduced FC during the “N-back” task in subjects with schizophrenia in contrast to healthy controls. These findings have introduced the notion of human large-scale networks underlying brain function during working memory. The FC correlational analyses do not allow for the inference of directions or weights of in functional connections. Thus, from FC findings it is not practical to draw inferences on neurobiological causal processing.

Studies, which applied DCM as a biophysical modeling approach to functional large-scale networks, showed that reduced EC findings could be interpreted in context of the NMDA receptor hypofunction model and the dysconnection hypothesis.

In summary, FC findings cannot be interpreted in context of the dopamine or glutamate hypothesis. For EC findings, the computational neuropsychiatric approach of modeling large-scale networks requires biophysically plausible networks, which are hypothesis-driven from neurobiological and cognitive neuroscience in subjects with schizophrenia. EC findings have been interpreted in the context of the glutamate hypothesis and the dopamine hypothesis.

#### Do the findings from computational neuropsychiatry lead to a gain in understanding of schizophrenia in comparison to the findings from clinical and cognitive neurosciences?

Functional connectivity findings from cognitive and clinical neuroscience have contributed to the understanding of schizophrenia as a cognitive brain disorder. The analysis of altered working memory at the level of large-scale networks has advanced our knowledge of cognitive function in humans. However, it is not wholly understood what altered FC during cognition neurobiologically means in schizophrenia. EC findings from computational neuropsychiatry, here specifically modeling functional large-scale networks with DCM, have shown indications of linkage between clinical network-based working memory (large-scale networks) and preclinical neurotransmitter modulation of cognitive function. Altered synaptic plasticity during working memory can be interpreted with dopaminergic and glutamatergic mechanisms. We emphasize that the interpretation of altered neurotransmitter circuits should be considered carefully because the DCM method is likely to underestimate the processing complexity in neurobiological circuits. Nonetheless, a strength of DCM lies in interpretation of altered synaptic plasticity based on the inference of mechanistic information.

The consideration of schizophrenia as a cognitive brain network disorder from computational neuropsychiatry offers a holistic view of schizophrenia. Computational neuropsychiatry seeks to bridge the gap between neurobiology and cognitive and clinical neurosciences in subjects with schizophrenia. It is hoped that this research will enhance our understanding of schizophrenia, clinical treatment, and improve outcome in people with schizophrenia.

### Future outlook and open questions

The reviewed findings in biophysical modeling of functional large-scale networks are promising. In order to reach the objective of predicting and improving clinical treatment in subjects with schizophrenia, longitudinal study designs, and the combination of subfields within computational neuropsychiatry should be pursued.

We consider computational neuropsychiatric research areas for the combination of biophysical modeling of functional large-scale networks and other computational (neuro)psychiatric approaches, which are of clinical relevance for subjects with schizophrenia, for example:

- Neurotransmitter systems
- Behavior
- Clinical symptoms
- Effects of antipsychotic medication
- Clinical outcome.

We suggest specific study designs, which may increase our understanding for developing clinical treatment for subjects with schizophrenia:

(i) Combination of biophysical modeling of functional large-scale networks with computation, for example:
  (a) Brain function and brain circuit model (12);
  (b) Brain function and behavior (141);
  (c) Brain function and effect of antipsychotic medication:
(ii) Combination of biophysical modeling of functional large-scale networks with multimodal neuroimaging study designs, for example:
  (a) FMRI and EEG/magnetoencephalography study designs;
  (b) FMRI and transcranial magnetic stimulation study designs (142);
  (c) FMRI and MRS study designs;
  (d) FMRI and PET study designs;
(iii) Combination of biophysical modeling of functional large-scale networks and computational modeling for the investigation of clinical (sub)groups, for example:
  (a) Associative learning (143, 144);
  (b) Machine learning approach (139, 145);
  (c) Reinforcement learning (109).

Findings of modeling functional large-scale networks contribute to shaping the understanding of schizophrenia as a cognitive brain network disorder. The combination of computational neuropsychiatric areas may bring researchers closer to the common long-term objectives of developing a diagnostic tool for schizophrenia along with the development of more effective treatments.

## Conflict of Interest Statement

Maria R. Dauvermann, Neil Roberts, Stephen M. Lawrie, and Thomas W. J. Moorhead have received financial support from Pfizer (formerly Wyeth) in relation to imaging studies of people with schizophrenia. Stephen M. Lawrie has done consultancy work for Roche Pharmaceuticals in connection with a possible new treatment for schizophrenia. Stephen M. Lawrie has also received honoraria for lectures, chairing meetings, and consultancy work from Janssen in connection with brain imaging and therapeutic initiatives for psychosis.

## Footnotes

^1^It is noted that other neurotransmitter circuits are interacting with dopaminergic and/or glutamatergic circuits such as serotonin and GABA (24, 29, 149).

^2^In this review, we focus on the “2-back” task [verbal “2-back”, (104); numeric “2-back”, (97)] to review/discuss brain function and PET findings of comparable experimental paradigms, psychological/ cognitive domains/components and activated brain regions.

^3^Exemplary studies on verbal fluency findings are presented.

^4^Reduced FC between the dorsal PFC and posterior PC.

^5^As one subfield within computational psychiatry.

^6^We consider DCM as the generative model approach as introduced in the seminal article by Friston et al. (18).

^7^It is noted, however, that the large-scale networks differed slightly from the previous study.

## Appendix

### Dynamic causal modeling

Dynamic causal modeling is general framework for model-based assessment of competing theories about neuronal circuits (18, 146). In particular, DCM is a generic Bayesian system identification technique, which allows for inference on “hidden” neurophysiological mechanisms that generated observed measures, such as blood-oxygen-level-dependent signal in functional magnetic resonance imaging (fMRI) or evoked responses measured with electroencephalography (EEG). The principle idea thereby is to formulate a simplified model of neuronal population responses (*z*) and combine this with a modality-specific forward model (λ) such that one can predict the measurement (*y*) that would arise from any particular neuronal circuit (18).

In DCM for fMRI, the dynamics of the neural states underlying regional BOLD response are modeled by a bilinear differential equation that describes how the neural states (*x*) change over time (*t*) as a function of endogenous inter-regional connections (matrix A), modulatory effects on these connections (matrix B), and direct (driving) inputs (matrix C) (Eq. A1) (18, 122). The endogenous connections represent coupling strengths in the absence of input u_j_ to the system, whereas the modulatory effects represent task-specific alterations in this connectivity.

(A1) $$f\left(x,u\right)=\frac{dx}{xt}=\left(A+\sum_{i=1}^{m}uB^{\left(j\right)}\right)x+Cu$$

The bilinear state equation has subsequently been extended by a non-linear term, where the modulation of connection strengths by experimental inputs is supplemented by direct modulation with neural activity in one or more regions (119). In other words, non-linear DCMs allow addressing how the connection between two neuronal units is gated by activity in other units. This is of particular interest as gating processes represent a key mechanism for many neurobiological processes and thus increasing the biological realism of non-linear compared to bilinear DCMs. To this end, compared with the bilinear state equation, the new term in the non-linear equations are the *D* matrices (Eq. A2), which encode how the *n* regions gate connections in the system.

(A2) $$f\left(x,u\right)=\frac{dx}{dt}=\left(A+\sum_{i=1}^{m}uB^{\left(j\right)}+\sum_{j=1}^{n}x_{j}D^{\left(j\right)}\right)x+Cu$$

### Bayesian model selection and bayesian model averaging

Bayesian model selection is an essential procedure of DCM studies as it can be used to test competing hypotheses (different DCMs) about the neural mechanisms generating the data. BMS rests on comparing the evidence of a predefined set of models (the model space). The model evidence is the probability of observing the empirical data, given a model, and represents a principled measure of model quality, derived from probability theory (147, 148). Concretely, it represents the mean predicted data under random sampling from the model’s priors or, alternatively, a principled measure of the balance between model fit and model complexity. A random-effects BMS approach has been suggested for group studies, which is capable of quantifying the degree of heterogeneity in a population while being extremely robust to potential outliers (20, 67). The probability that one model is more likely than any other model, given the group data, can be expressed by the exceedance probability (φ*_k_*) of each model:

$$\begin{array}{llll} \exists k & \in \left\{1\ldots k\right\},\forall j\in \left\{1\ldots k|j\neq k\right\}\;:\; & & \;\\ \varphi_{k} & =p\left(r_{k}>r_{j}|y;a\right)\; & & \; \end{array}$$

After inference on the most likely network architecture underlying a specific neural process, one can compare the parameter estimates of the most likely model obtained from BMS (winning model) for between-group inferences. However, statistical comparison of model parameter estimates across groups is only valid if those estimates stem from the same model. Given that different models may be found to be optimal across groups, Bayesian model averaging (BMA) has been recommended as standard approach for clinical DCM studies (146). BMA averages posterior parameter estimates over models, weighted by the posterior model probabilities (148). Thus, models with a low posterior probability contribute little to the estimation of the marginal posterior. In brief, BMS and BMA are central components of DCM studies to infer on neural mechanisms at the neural system level and on specific model parameters across groups, respectively (146).

In non-linear DCM analysis, the connection strengths between selected nodes are assessed for activity-dependent modulation of the reciprocal neuronal projections by the introduction of gating mechanisms. Non-linear DCM is applied to the models identified as winning models from the application of bilinear state equation. The bilinear model and the non-linear models differ only in the introduction of gating mechanisms such as a parametric response in the tested functional task. Such gating mechanisms are applied to nodal connections, which are expected to explain the variation in subject response to the functional task. The appropriate placement of the gating input is assessed through the application of model space partitioning and family inference. The exceedance probabilities of the models are compared and the non-linear models, which provide higher exceedance probabilities than the bilinear models are identified as winning models.

## Abbreviations

AC/ACC, anterior cingulate/anterior cingulate cortex; ARMS, at-risk mental state; BMS, Bayesian model selection; BOLD response, blood-oxygen-level dependent response; D_1_ receptor, D_1_ subtype of the dopamine receptor; D_2_ receptor, D_2_ subtype of the dopamine receptor; D_2/3_ receptors, D_2/3_ subtype of the dopamine receptor; DCM, Dynamic Causal Modeling; DMN, default-mode network; DLPFC, dorsolateral prefrontal cortex; EC, effective connectivity; EST, subjects with EST; FC, functional connectivity; FEP, subjects with first episode psychosis; FES, subjects with first episode schizophrenia; GABA, γ-aminobutyric acid; GBC, global-based connectivity; HR, subjects at high risk of schizophrenia; HR+, subjects at high familial risk of schizophrenia with transient psychotic symptoms; HR−, subjects at high familial risk of schizophrenia without transient psychotic symptoms; HRill, subjects at high familial risk of schizophrenia who subsequent to scanning developed schizophrenia; HSCT, Hayling sentence completion task IFG inferior frontal gyrus; MFG, middle frontal gyrus; MRS, magnetic resonance spectroscopy; NMDA, *N*-Methyl-d-aspartate acid; PC, parietal cortex; PET, positron emission tomography; rCBF, regional cerebral blood flow; SPECT, single-photon emission computed tomography; SPL, superior parietal lobe; STG, superior temporal gyrus.
